# Sources of metal pollution in the urban atmosphere (A case study: Tuzla, Istabul)

**DOI:** 10.1186/s40201-015-0224-9

**Published:** 2015-11-19

**Authors:** Abdullah Aksu

**Affiliations:** Department of Chemical Oceanography, Institute of Marine Science and Management, Istanbul University, Vefa 34134-00, Istanbul, Turkey

**Keywords:** Atmospheric pollution, Metal, İstanbul, Tuzla, Enrichment Factor (EF)

## Abstract

In this study, İstanbul-Tuzla Region atmosphere was selected as the working area for atmospheric pollution. Although the study area seems too local, this region contains shipyards and shipyard-related side product industries. It is also surrounded by aluminum (ASSAN) and copper (SARKUYSAN) facilities and tannery organized industrial district. For determined the atmospheric inputs, the sample collection was carried out as monthly in 2010. Particulate matter was filtered from aerosols via a high volume air sampler. The collected 46 ambient air samples were analyzed for Cr, Fe, Cu, Zn, Cd, Pb and Al using Atomic Absorption Spectrometry (AAS), Flame unit. Additionally, the volume of the air was drawn and meteorological data recorded.

Average individual heavy metal concentrations were found as Cd (0.06 ng/m^3^) < Cr (0.09 ng/m^3^) < Zn (0.21 ng/m^3^) < Pb (0.23 ng/m^3^) < Cu (0.48 ng/m^3^). The concentrations of crustal elements Fe and Al were changed between 5.48 ng/m^3^, 74 ng/m^3^ and 14 ng/m^3^, 284 ng/m^3^ respectively during the sampling period. Except Cr and Fe anthropogenic contribution was seen on the concentrations of Zn, Cu, Pb and Cd in an increasing order. While the crustal element Fe was not show an appreciable change in concentration, but the Al concentration was display an important change in concentration depending on the wind transportation.

## Introduction

Various chemicals are emitted into the air from both natural and man-made (anthropogenic) sources. The quantities may range from hundreds to millions of tonnes annually. Natural air pollution stems from various biotic and abiotic sources such as plants, radiological decomposition, forest fires, volcanoes and other geothermal sources and emissions from land and water. These result in a natural background concentration that varies according to local sources or specific weather conditions [1]. The emission of toxic substances in the environment has been spread from industrialized countries. Many industrial plants and also heavy traffic may produce heavy metal into atmosphere. Traffic pollutants include potentially toxic metals for health like lead, cadmium and zinc [2, 3]. Migon et al. [4] indicated after the implementation of antipollution policies on automotive Pb concentration levels decreased by 82 %. The changes in life style increases the levels at which trace metals are added in soil, water and air from anthropogenic sources [5, 6]. Heavy metal pollution is potentially a persistent problem all over the world in an increasing order [7].

Emissions from traffic contain many toxic heavy metals such as; Pb, Cd and Zn [2]. Meteorological conditions and local sources have an important role on trace element concentrations. Airborne particles are important carriers of metals [8].

In recent decades, major efforts have been made to reduce air pollution in the European Region [1]. So a considerable interest has been given to particulate matter (PM) of aerosols due to their impacts on visibility, human health, plants, aquatic life and materials [8–10].

While the industrialization and human activities intensify the emission of various pollutants into the environment and introduce various harmful substances into the atmosphere [3].

A few decades ago the study are Tuzla region in İstanbul was a coastal recreation area. The industrialization of the Tuzla region had been increased rapidly. Especially shipyards and shipping industry were growth in the region. Such areas were classified “transitional urban areas” had rapid population growth between 1975 and 2005 but tapered projected growth between 2005 and 2015 such as İstanbul, Turkey [11]. The aim of this study is firstly to search the seasonal change of metals Cr, Fe, Cu, Zn, Cd, Pb and Al of concentrations as inorganic pollutants and secondly to find their sources and anthropogenic contributions for the first time for this region.

## Material and methods

### Sampling

**Fig. 1:**
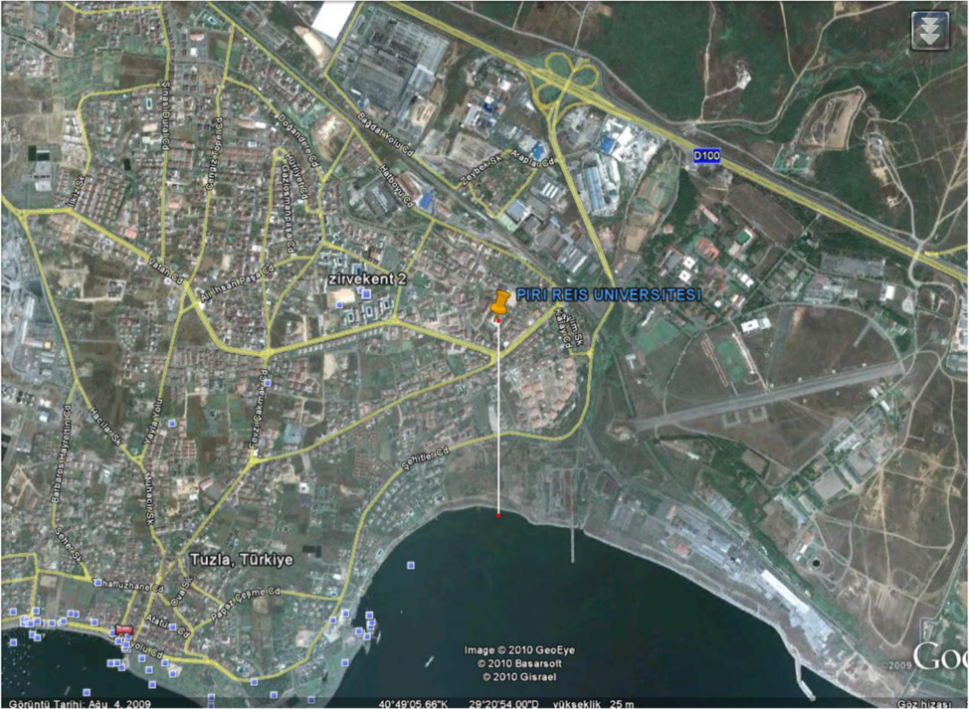
Location of sampling area (Google Earth)

The sampling area is located in Turkey/İstanbul, TUDEV-Piri Reis University Campus (40^0^ 49′ 05 66″N and 29^0^ 20′54 00″E) at a height of 22 m (Fig. 1). The sampling point is faraway 0.81 km from the Marmara Sea. A High Volume Air Sampler (HVAS) was fixed at the top of the University Building roof to carry out the aerosols sampling.

The sample collection was carried out at 2010. The filter papers divided into two parts; one halves were saved in plastic bag separately for metal analyses. Five samples were taken for February, March, April, May, June, July, August, September and October. Only one sample was obtained for December and this value assumed as the average of the month. The volume of the air was drawn and meteorological data recorded.

### Analytical procedures

#### Particulate matter

Whatman glass fiber filter papers having a pore size of 0,1 μm are dried for 48 h in a desiccator and weighted before and after the sampling. So the daily concentrations of particulate matter with the size > 0.1 μm of collected over the filter paper were found.

### Metal analyses

The halves of the filter papers put into the teflon (PTFE) tubes. A strong acid mixture of 5 ml HNO_3_, 3 ml HClO_4_ and 1 m l HF is added to the tubes to provide the decomposition metals at 180 °C in closed digestion unit (micro-wave) during one hour. Then diluted to a definite volume and then the analyses of Al, Fe, Pb, Zn, Cd and Cr are accomplished at Atomic Absorption Spectrometry (AAS), Flame unit. Duplicate metal analysis results are given in Table 1. Confidence interval = 1.96 × standard deviation/mean.

**Table 1:** The sensitivity of duplicate metal analysis

Metal	Mean-st.deviation	Confidence interval
Cd	0.00735 ± 0.00021	0.0056
Pb	0.1153 ± 0.00113	0.019
Cu	0.34365 ± 0.01463	0.083
Cr	0.00825 ± 0.00007	0.0166
Zn	0.2044 ± 0.00806	0.077
Fe	13.3526 ± 0.32682	0.0479
Al	47.6121 ± 1.02968	0.0423

## Results

### Particulate matter

**Fig. 2:**
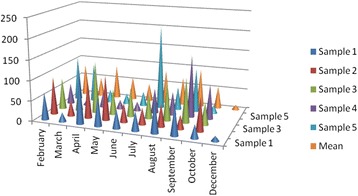
Daily PM concentrations (μg/m^3^)

The air quality standard adapting to US EPA NAAQS (http://www.epa.gov/air/criteria.html) for PM_10_particles in Turkey is 150 μg/m3 and required to decrease the concentration below 60 μg/m3until 2014 for human health [12]. As a result of the normal day-to-day variation in PM_10_ concentrations, 24-h averages of 100 μg/m^3^ are regularly exceeded in many areas in Europe, especially during winter inversions. The effects have been observed at annual average concentration levels below 20 μg/m^3^ (as PM_2.5_) or 30 μg/m^3^ (as PM_10_). For this reason, no guideline value for long-term average concentrations is recommended [1]. In Turkey, it is required to reach these limit values at 2019 [12]. The range of PM (pore size >0.1 μm) concentrations measured at Tuzla region during the sampling period was 12.22 μg/m^3^-209 μg/m^3^ with the average value of 67.90 μg/m^3^. The daily PM concentrations in each month were shown on Fig. 2. The tendencies in increase at PM concentrations were seen on from July to October in some measurements.

Cong et al. [13] studied with two filter paper simultaneously, one with polytetrafluoroethylene, PTFE, 0.5 μm, 47 mm) and the other polycarbonate (Nucleopor, Coaster,) filters with the pore size of 1.5 %. In the study the results were found in between 2.02-6.34 μg/m^3^. Braga et al. [14] measured the concentration (μg/m^3^) ranges between HV PM_10_ in 2.80-40.10 and Dichotomous (fine) in 12 0.46-16.02 and Dichotomous (coarse) in 1.51-24.42.

Table 2 shows the meteorological conditions and the measured PM concentrations (μg/m^3^) ialong the sampling periods (Fig. 2). This may not be enough to explain all the measurement events, since the initial measurements generally depend on the previous days’ meteorological conditions. It is necessary to develop a detailed study to explain the relationship between the meteorological parameters and the PM concentrations in the aerosol such as wind transportation. Although there are some deficiencies to explain the relationships between meteorological conditions and PM concentrations, this table will help to see some variations on PM concentrations. The weak correlations are found between PM concentrations with average temperature, relative humidity and average wind speed are 0.15, 0.08 and 0.18 respectively. To observe any relationship between meteorological conditions PM concentrations all parameters could be considered as a whole previous or sampling period. For instance, from Table 2, it is seen that the low concentrations were generally occurred at low temperatures and rainy days, e.g. December Sample No. 1 (1.22 μg/m^3^) and March Sample No. 4 (12.87 μg/m^3^). The high concentrations were seen at dry air and when humidity values are relatively low, e.g. July Sample No. 5 (209 μg/m^3^) and September Sample No. 4 (154 μg/m^3^). As an exceptional case, a dust concentration (0.25-0.50 mg/m^3^) had been occurred at February 17 from Sahara Desert and continued a few days causing to decrease the visibility to 10–6 km (www.meteorgov.tr). This effect was seen on the PM concentrations on Sample No. 2 (93.11 ng/m^3^) and Sample No. 3 (78.46 ng/m^3^) even if the duration was rainy. After a dry period, the concentration increase was observed on the fifth sample depending on wind transportation. In Turkey, the wind systems generally cause to flow of air from west to 22 east [15, 16].

**Table 2:** Meteorological parameters in the year 2010

Sampling date/duration	No	Average temp. (°C)	Wind-speed (Ave-Max) (km/h)	Wind direction	Precipitation (mm)	Relative humidity (%) (Ave-Max)
12.02.2010/(10:00)-13.02.2010/(09:30)	1	11	12-33	SW	0.2	72-83
16.02.2010/(16:00)-17.02.2010/(09:00)	2	7	7-15	NE	0.6	61-70
17.02.2010/(17:00)-18.02.2010(09:00)	3	12	10-23	SW	0.4	52-70
18.02.2010/(17:00)-19.02.2010/(09:30)	4	7	8-20	SW	-	80-90
22.02.2010/(17:00)-23.02.2010/(09:00)	5	8	6-18	S-SW	-	58-71
11.03.2010/(17:00)-12.03.2010/(09:00)	1	8	16-28	N-SW	showery	81-84
15.03.2010/(17:00)-16.03.2010/(09:00)	2	6	11-20	N-S	-	66-59
16.03.2010/(17:00)-17.03.2010/(09:00)	3	4	15-32	S	0.4	59-63
17.03.2010/(17:00)-18.03.2010/(12:00)	4	2	8-17	N-S	0.2	48-63
18.03.2010/(17:00)-19.03.2010/(09:00)	5	6	6-15	N-E	-	48-51
15.04.2010/(18:00)-16.04.2010/(10:30)	1	14	5-15	N-SW	-	86-90
16.04.2010/(18:00)-17.04.2010/(19:00)	2	14	20-32	N	-	71-57
19.04.2010/(09:30)-20.04.2010/(09:30)	3	14	10-20	N-S	0.6	71-100
20.04.2010/(17:30)-21.04.2010/(17:00)	4	13	15-35	N	0.8	80-100
21.04.2010/(17:00)-22.04.2010/(09:30)	5	13	11-22	W-SW	-	59-100
12.05.2010/(17:00)-13.05.2010/(09:00)	1	21	7-15	N-E	0.6	60-85
14.05.2010/(17:00)-15.05.2010/(17:00)	2	20	10-20	S-E	-	55-60
17.05.2010/(17:00)-18.05.2010/(17:00)	3	18	22-32	S-N	-	60-70
18.05.2010/(17:00)-19.05.2010/(16:00)	4	16	12-17	S-W	-	57-70
20.05.2010/(16:00)-21.05.2010/(13:00)	5	17	8-22	S-N	-	40-57
18.06.2010/(17:00)-19.06.2010/(18:00)	1	25	17-26	N	8	62-59
19.06.2010/(18:00)-20.06.2010/(16:00)	2	26	9-20	N-S	8	60-70
20.06.2010/(16:00)-21.06.2010/(09:00)	3	24	10-20	N-S	-	70-80
21.06.2010/(17:00)-22.06.2010/(17:00)	4	22	13-39	S	-	54-57
22.06.2010/(17:00)-23.06.2010/(17:00)	5	23	12-24	E-SW	-	54-65
19.07.2010/(18:00)-20.07.2010/(17:00)	1	26	15-37	N	-	62-89
20.07.2010/(17:00)-21.07.2010/(17:00)	2	25	11-24	N	-	69-83
21.07.2010/(17:00)-22.07.201/(18:00)	3	27	15-30	N	-	72-94
22.07.2010/(17:00)-23.07.2010/(17:00)	4	29	15-26	N	-	63-89
23.07.2010/(17:00)-24.07.2010/(09:00)	5	27	11-18	N-S	-	67-90
09.08.2010/(17:00)-10.08.2010/(11:00)	1	25	15-35	N	exist	75-89
10.08.2010/(17:00)-11.08.2010/(17:00)	2	30	14-39	N	-	70-89
11.08.2010/(17:00)-12.08.2010/(17:00)	3	28	14-28	N	-	67-83
12.08.2010/(17:00)-13.08.2010/(17:00)	4	39	9-16	N-S	-	69-94
13.08.2010/(17:00)-14.08.2010/(11:00)	5	30	9-22	Variable	-	69-50
20.09.2010/(17:00)-21.09.2010/(17:00)	1	22	14-39	N-NW	-	75-100
21.09.2010/(17:00)-22.09.2010/(17:00)	2	20	27-41	N-NE	-	70-88
22.09.2010/(17:00)-23.09.2010/(17:00)	3	21	21-33	N	-	58-63
26.09.2010/(17:00)-27.09.2010/(17:00)	4	26	8-20	S-SE	-	60-78
28.09.2010/(17:00)-29.09.2010/(17:00)	5	22	10-18	S-SW	-	61-78
13.10.2010/(17:00)-14.10.2010/(17:30)	1	17	7-15	N-S	5	87-94
14.10.2010/(17:00)-15.10.2010/(14:00)	2	17	13-18	NE	9	90-100
16.10.2010/(19:00)-17.10.2010/(19:00)	3	18	9-18	N-NE	2	90-100
17.10.2010/(18:00)-18.10.2010/(18:00)	4	18	5-13	S-SW	-	81-94
18.10.2010/(18:00)-19.10.2010/(19:00)	5	19	9-17	S	2	70-88
13.12.2010/(17:00)-14.12.2010/(17:30)	1	4	15-26	N	8	62-87

### Metals

**Fig. 3:**
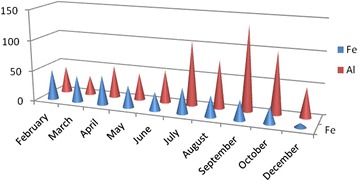
Monthly average concentrations (ng/m^3^) of Al and Fe

In this study metals are evaluated into two groups as crustal metals Al and Fe and non-crustal metals Cr, Cu, Zn, Cd and Pb. While the Al and Fe monthly average concentrations are shown in Fig. 2a, the others are shown Fig. 2b. Al concentrations begin to increase from July and reach a peak at September, then the concentration decreases at cold months (Fig. 3a). This situation shows some similarities with PM concentration changes (Fig. 2). Fe does not show any appreciable change in monthly average concentrations except December (Fig. 3a).

**Fig. 4:**
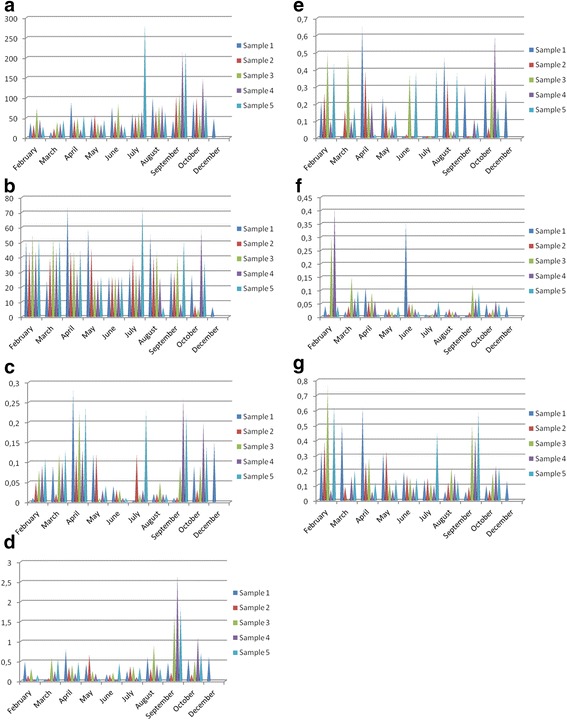
**a**. Daily concentration changes of Al concentration (ng/m^3^). **b**. Daily concentration changes of Fe concentration (ng/m^3^). **c**. Daily concentration changes of Cr concentration (ng/m^3^). **d**. Daily concentration changes of Cu concentration (ng/m^3^). **e**. Daily concentration changes of Zn concentration (ng/m^3^). **f**. Daily concentration changes of Cd concentration (ng/m^3^). **g**. Daily concentration changes of Pb concentration (ng/m^3^)

From Fig. 4b, it can be concluded that, while the monthly average concentrations of Cr, Zn, Cd, Pb show some extremums at different months, Cu shows an increase in average concentrations from beginning July reach to a maximum at September, then decreases. Copper concentration changes also show some similarities with PM and Al concentrations.

The daily metal concentrations of metals are given in Table 2 and Fig. 4a-g.

### Crustal metals

In this study, aluminum concentration changes between 14 ng/m^3^ and 248 ng/m^3^ with an annual average of 71.13 ng/m^3^ (Table 2, Fig. 4a). The highest concentration is found in the 5^th^ sample of July. The PM concentration also has the highest concentration that sampling period. The correlation between Al and PM was found as r = + 0.64 (Table 3). The lowest Al concentration is found in March, Sample No. 1as14 ng/m^3^. This sampling duration corresponds a rainy period. The second low Al concentration is found in a rainy period in April Sample No.4 as 21 ng/m^3^. The increases in concentrations in September could be explained that there wasn’t any precipitation. In October, the period was rainy and high relative humidity. So Al could be hold in the aerosol by high humidity.

**Table 3:** Correlation coefficients among metals and meteorological data

	Fe	Cu	Zn	Cd	Pb	Al	PM	Temp.	R.Hum.	Wind speed
Cr	0.30	**0.54**	0.29	0.15	0.47	0.49	**0.51**	0.21	0.10	0.38
Fe	-	0.03	0.40	0.25	0.44	0.23	0.48	0.19	0.08	0.24
Cu		-	0.05	0.00	0.37	**0.62**	0.47	0.20	0.008	0.10
Zn			-	0.08	0.25	0.12	0.33	0.14	0.34	0.37
Cd				-	0.22	0.05	0.08	0.27	0.04	0.06
Pb					-	0.34	**0.56**	0.14	0.05	0.25
Al						-	**0.64**	0.43	0.17	0.08

In İstanbul (Tuzla region), iron concentration changes between 5.48 ng/m^3^ and 74 ng/m^3^with the average value of 37.22 ng/m^3^ (Table 2 and Fig. 4b). Fe concentrations are generally low at rainy sampling periods e.g., August October Sample No.2 and Sample No. 3 are 7.66 ng/m^3^ and 5.48 ng/m^3^. The high concentrations were observed at dry periods, e.g. both April Sample No.1 and July Sample No.5 was measured as 74 ng/m^3^. Fe concentrations were low in some measurements at August, October and December due to washing out of precipitation. The correlation between Fe and PM found as r = + 0.48.

### Non-crustal metals

In this study, chromium concentration range was in between 0.004 ng/m^3^ and 0.28 ng/m^3^, with an average value of 0.089 ng/m^3^ (Table 2, Fig. 4c). The highest concentrations were observed at rainy and high relative humidity sampling days e.g., April Sample No. 1 and sample No. 3 were 0.28 ng/m^3^ and 0.23 ng/m^3^, July Sample No. 5 was 0.23 ng/m^3^, September Sample No. 4 and Sample No.5 were 0.26 ng/m^3^ and 0.22 ng/m^3^. The lowest chromium concentration was found in July Sample No 1. (0.004 ng/m^3^). There wasn’t any precipitation and the relative high humidity value was low. Some low concentrations were observed at heavy rains and low relative humidity sampling periods e.g. June sample No 4 and 5 the concentration was measured as 0.1 ng/m^3^. The correlation between Cr and PM was found as r = +.0.51 (Table 3).

The high Cr concentrations was observed when the prevailing wind directions are generally S and SW. Taşdemir et al. [8] was stated the leather industry caused in increase particularly on Cr levels depending on wind direction in Bursa In the study the correlation of Cr with average wind speed was found as r = + 0.38 that was the highest wind speed correlation (Table 3). In the study the correlation of high concentrations of chromium with average wind speed was found 6 as r = + 0.53.

In Tuzla region, the annual copper concentration was found as 0.48 ng/m^3^within the range of 0.04 ng/m^3^ and 2.68 ng/m^3^ (Fig. 4d). The highest concentrations were observed at September. During the sampling period, there weren’t any precipitation and the humidity values were high. The seasonal concentration fluctuations of Cu similar to Al. The correlation of Cu with Al was r = + 0.62 (Table 3).

In the study area, zinc concentration ranges were in between 0.01 ng/m^3^ and 0.65 ng/m^3^ with an average concentration of 0.18 ng/m^3^. The highest concentrations were measured at April and October, when the relative humidity percentages were high (Fig. 4e). The highest correlation was found between Zn and relative humidity percentage was 0.34 and the correlation between high concentrations of Zn with the precipitation was found as r = + 0.58 (Table 3).

In Tuzla aerosol, cadmium concentration range was 0.001 ng/m^3^- 0.41 ng/m^3^ with an average of 0.063 ng/m^3^ (Fig. 4f). The high concentrations were observed two times at February and one times at June. These measurements correspond to rainy periods. The correlation between Cd and PM was r = + 0.48 (Table 3). Cizmecioğlu and Müezzinoğlu [17] found that the samples taken during rain incidents following elongated dry periods were high. And also parallel results were found by [18] who found high concentrations after a dry period and when rain continued for several days, concentrations were lower in rain in Amman, Jordan.

In Tuzla region lead concentration range was 0.01 ng/m^3^ – 0.77 ng/m^3^ with a mean of 0.23 ng/m^3^ (Fig. 4g). Higher concentrations of Pb were seen on high PM concentration periods. The correlation between Pb and PM was found as r = + 0.56 (Table 3).

## Discussion

Correlation coefficients among metals and meteorological data of this study are given in Table 3. The highest value (r = + 0.64) was found between Al and particulate matter contents in atmospheric aerosols. Additionally, high correlations were observed between Al and Cu contents, particulate matter and Pb, particulate matter and Cr amounts. The results are show the terrestrial origins on this metal distributions via atmospheric tranport. In contrast, the low correlation coeffecient values are indicate the different origins and factors on atmospheric metal transport.

Table 4 is prepared to compare this study results with other studies. Except Terra Nova Bay [19] the Cr and Fe concentrations of the region was very low from the other studies. The Al concentration of the region was higher than Mt. Qomolangma [13] and Terra Nova Bay [19] but the Al concentration of the Ankara having a continental climate [9] and West and East Black Sea aerosols were very high [16]. Zn and Cd measurements are similar to Terra Nova Bay [19]. Cu concentration was similar to Mt. Qomolangma [13] and Terra Nova Bay [19]. It could be concluded that out of Cr, Fe and Al, Tuzla region measurements show some approximate values with terra Nova Bay.

**Table 4:** Mean and max-min values of metal concentrations (ng/m^3^) measuredat this study and comparison with other studies

Metal	This study			Mt. Qomolangma^a^			Ankara^b^	Black Sea^c^		TerraNova Bay^d^
								West	East	
	Mean	Min	Max	Mean	Min	Max	Mean	Mean	Mean	Mean
Cr	0.089	0.004	0.28	3.0	2.3	12	3.2	9.0	8.3	0,043
Fe	37.22	5.48	74	61	28	89	100	420	420	2.31
Cu	0.48	0.04	2.68	0.31	0.21	0.69	-	-	-	0.422
Zn	0.18	0.01	0.65	1.4	0.81	3.3	16	46	26	0.081
Cd	0.063	0.001	0.41	-	-	-	0.11	-	-	0.016
Pb	0.23	0.01	0.77	0.43	0.30	0.59	71	-	-	-
Al	71.13	14	248	55	34	85	110	540	330	2.08

^a^Cong et al., [13]: Mt. Qomolangma,Everest, China – Atmospheric aerosols in May-June 2005

^b^Yatin et al., [9]: METU Campus, Ankara, Turkey – Atmospheric aerosols in February-June 1993

^c^Güllü et al., [16]: West and East Black Sea, Turkey – Over the atmosphere near sea level, June, August-September 1988

^d^Toscano et al., [19]: TerraNova Bay, Antartic – Coastal site aerosols, Summers of 2000–2001 and 2001–2002

In general, most people take in very little aluminum from breathing. Levels of aluminum in the air generally range from 0.005 to 0.18 micrograms per cubic meter (μg/m^3^), depending on location, weather conditions, and type and level of industrial activity in the area. Most of the aluminum in the air is in the form of small suspended particles of soil (dust). Aluminum levels in urban and industrial areas may be higher and can range from 0.4 to 8.0 μg/m^3^ (http://www.atsdr.cdc.gov/toxprofiles/tp22-c1.pdf). The Occupational Health and Safety Administration (OSHA) has limited workers’ exposure to aluminum in dusts to 15 mg per cubic meter (mg/m^3^) (total dust) and 5 mg/m^3^ (respirable fraction) of air for an 8-h workday, 40-h workweek (http://www.atsdr.cdc.gov/tfacts22.pdf). Aluminum is released to the environment by both natural processes and anthropogenic sources. Because of its prominence as a major constituent of the earth’s crust, natural processes far exceed the contribution of anthropogenic releases to the environmental distribution of aluminum [20]. Sekhavatjou et al. [21] indicate that the Al level in industrial and heavily congested traffic sectors is higher than other areas. Coal burning, electroplating and paint are the source of Al [22]. Iron is not considered to cause harmful health effects in general; toxicological and epidemiological literature is limited. ATSDR [23] used a modification of the dose equation (dose = concentration × intake rate) to calculate a daily consumption from exposure to iron in soil. Exposure to the maximum concentration of iron collected from a residential area (31,500 ppm). The National Academy of Sciences’ (NAS’s) dietary reference intake (DRI) for children 1 to 3 years old is 7 mg/day [24]. The median daily intake of dietary iron is roughly 11 to 13 mg/day for children 1 to 8 years old and 13 to 20 mg/day for adolescents 9 to 18 years old [23, 24]. (http://www.atsdr.cdc.gov/HAC/pha/oakRidge013107-TN/Final_Current_Screening_1-07_508.pdf. The vehicular activities cause to increase the level of Fe in the atmosphere [25].

The concentration level of Fe is highly influenced by the industrial activities and traffic of vehicle [21]. Chromium is ubiquitous in nature. Available data, generally expressed as total chromium, show a concentration range of 5–200 ng/m^3^. There are few valid data on the valency and bioavailability of chromium in the ambient air [1]. Cr is emitted in the atmosphere due to coal and oil combustion, especially diesel-fed vehicles, refuses incineration [26]. The Occupational Safety and Health Administration (OSHA) requires that levels of copper in the air in work places not exceed 0.1 mg/m^3^ copper fumes per cubic meter of air (0.1 mg/m^3^) and 1.0 mg/m^3^ for copper dusts [27]. The main source of Cu in the atmosphere is diesel engines, unleaded gasoline is the other source [28]. The other sources of the atmosphere are Cu-containing fungicides, metal working factories and electroplating materials [29]. The rural levels of airborne copper represented regional background levels in urban study sites with only episodic increases, depending on wind speed and direction and location relative to local point sources. In one urban study site (East St. Louis), smelters are the primary source of copper. Copper depositional fluxes follow an exponential decay as one transition from urban to rural settings [30]. Soil is not the major source of copper in cities or nearby rural soils, but is the predominant source for copper in the atmosphere over more remote areas [27, 31]. In general, levels of zinc in air are relatively low and fairly constant.

Average levels of zinc in the air throughout the United States are less than 1 microgram of zinc per cubic meter (μ g/m^3^) of air, but range from 0.1 to 1.7 μg/m^3^ in areas near cities [12, 32]. Zn is used in lubricating oil [30, 33] and Zn is also released from vehicular activities such as tire wear. A WHO air quality guideline for cadmium of 5 ng/m3 has been recommended in order to prevent any further increases in cadmium levels in agricultural soils [1]. By the end of the period studied, concentrations over western and central parts of Europe declined, ranging from 0.1 to 0.3 ng/m3 [34]. In Turkey, the limit value is 5 ng/m3. And it is planned to decrease the level 2 ng/m3 at 2020. Cd is originated from vehicular activities [33]. Average air lead levels are usually below 0.15 μg/m^3^ at nonurban sites. Urban air lead levels are typically between 0.15 and 0.5 μg/m^3^ in most European cities. Additional routes of exposure must not be neglected, such as lead in dust, a cause of special concern for children [1]. By 2003, the concentrations in air had decreased markedly and were within 2–15 ng/m^3^ over most of Europe [34]. In Turkey, Pb limit value is 2 μg/m^3^, and it is required to decrease the value to 1 μg/m^3^ at 2019 [12]. Pb concentration levels in air are strictly depended on distance of the emission sources [35]. After the implementation of antipollution policies on automotive, Pb levels were decreased in the atmosphere by 82 % [4]. To improve the antiknock property, Pb is still being introduced in different forms to fuels in many countries [5].

Table 5 shows the mean and max-min values of EF_c_ values of heavy metals and their EF_c_/Al correlations. Non-crustal contributions on heavy metal concentrations of the region aerosol are observed as Cd > Pb > Cu > Zn. Migon and Nicolas [36] had been found the importance of the recycling component decreases in the sequence Cd > Pb > Cu > Zn in the North-western Mediterrenean atmosphere. (These metals are also found in high levels in some studies [8, 37].

**Table 5:** Mean and max-min. EF values of heavy metals according to Al and EF/Al correlations

EF-Al correlations	Cr	Fe	Cu	Zn	Cd	Pb
Mean	0.13	0.87	11.06	4.67	804.11	30.64
Max.	0.54	2.2	25.35	18.27	9730	243
Min.	0.005	0.06	1.43	0.11	8	4
r (EF-Al)	0.30	0.59	0.05	0.34	0.17	0.30

The enrichment factor (EF_crust_) is used to define the origin of the metals in aerosol [38, 39]. EF_c_ (enrichment factor from crust to air) = (C_x_/C_Al_)_air_/C_x_/C_Al_)_crust_, here; (C_x_/C_Al_)_air_ : the concentration ratio of the element X and Al in the measured air. (C_x_/C_Al_)_crust_ : the concentration ratio of the element X and Al in the crust. The elements have EF_c_ values which range between 1 and 10, indicating that they are present in the aerosol in roughly crustal proportions. Except Cr, Fe the other heavy metal concentrations are independent of Al concentrations. So, it can be said that their source are non-crustal [9]. Anthropogenic contributions on heavy metal concentrations of the region aerosol are observed as Cd > Pb > Cu > Zn in a decreasing order. The EF_c_ values of Cd 8–9730 with an average of 804.11 indicates that the high anthropogenic contribution. EF_c_ values of Pb correspond to a range of 10–66, with an average value of 30.64 so the anthropogenic contribution could be considered besides the natural occurrence. The EF_c_ range of Cu 1.43-25.35 with the average of 11.06 indicate that natural source was all, but also anthropogenic contribution was so high. The EF_c_ range of Zn was 0.11-18.27 with an average of 4.67 indicated that there were some anthropogenic contribution.

**Fig. 5:**
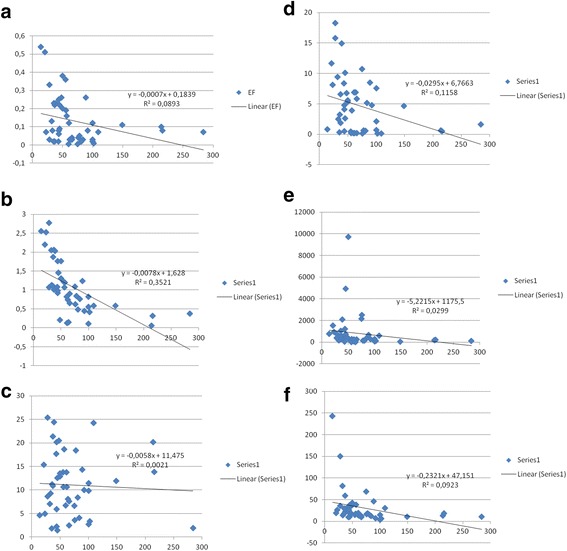
**a**. Cr enrichment factor versus Al concentration (EF_c_-Al)r = + 0.30. **b**. Fe enrichment factor versus Al concentration (EF_c_-Al)r = + 0.59. **c**. Cu enrichment factor versus Al concentration (EF_c_-Al)r = + 0.05. **d**. Zn enrichment factor versus Al concentration (EF_c_-Al) r = + 0.34. **e**. Cd enrichment factor versus Al concentration (EF_c_-Al)r = + 0.17. **f**. Pb enrichment factor versus Al concentration (EF_c_-Al)r = + 0.30

To observe the non-crustal (anthropogenic) contribution on measured metal concentrations, their EF_c_-Al diagrams are prepared and shown in Fig. 5d. From figures it is seen that most of the elements are independent of Al concentrations when Al concentration > 67.50 ng/m^3^. However, EF_c_ of these elements decreases with increasing Al concentration at lower Al concentrations which is a typical behavior of elements with non-crustal sources [9].

## Conclusions

Based on the results of this research, the concentrations of Cr and Fe in the ambient air of İstanbul (Tuzla region) indicate that their presence in the aerosol comes from (depends on) crustal origin. The enrichment factor of Tuzla aerosol were found Cd > Pb > Cu > Zn in a decreasing order. It may not be possible to make any generalization about the episodic concentration changes of the metals due to their different origin. But, almost all the metal concentrations are low at June paralleling to PM concentrations. To determine the emission sources of these metals, it is recommended to do more detailed and comprehensive study. The planned study must include wind transportation from industrial smokestacks and organized industrial areas located near the residential location in Tuzla region. For this reason all the meteorological parameters especially wind direction data could be available locally. When metals are introduced to atmosphere, they are passing through water and soil. Then, they are taken by living organisms via ingestion, inhalation and skin absorption. In conclusion, this work results are a basic source for future studies and the study will be unique and a useful model for other industrial areas to determine pollution in a large range entirely on account of having examined inorganic pollutants.
